# Development of a Mycoinsecticide Bait Formulation for the Control of House Flies, *Musca domestica* L.

**DOI:** 10.3390/insects11010047

**Published:** 2020-01-09

**Authors:** Dalton Baker, Steven Rice, Diana Leemon, Rosamond Godwin, Peter James

**Affiliations:** 1 Queensland Alliance for Agriculture and Food Innovation, The University of Queensland, Brisbane 4072, Australia; p.james1@uq.edu.au; 2 Agri-Science Queensland, Department of Agriculture and Fisheries, Brisbane 4102, Australia; Steven.Rice@daf.qld.gov.au (S.R.); Diana.Leemon@daf.qld.gov.au (D.L.); 3 Australian Banana Growers’ Council, Brisbane 4106, Australia; Rosie@abgc.org.au

**Keywords:** biological control, *Metarhizium anisopliae*, biopesticide, entomopathogenic fungi

## Abstract

The control of house flies, *Musca domestica* (L.), currently relies on the use of chemical insecticide spray and bait formulations. Entomopathogenic fungi, such as *Metarhizium* *anisopliae*, may provide an alternative to these products. This study aimed to develop and evaluate a mycoinsecticide bait formulation containing a virulent *M. anisopliae* isolate. Five *M. anisopliae* isolates were screened against *M. domestica* and isolate M16 was selected for bait development. Bait formulations containing a variety of additives, including (Z)-9-tricosene, were tested for their ability to increase fly visitation. A bait formulation containing *M. anisopliae* and skim milk powder was found to have the highest house fly visitation and was subsequently compared to a conventional chemical bait in an efficacy assay. The chemical bait (0.5% imidacloprid) caused faster mortality than the mycoinsecticide bait, however, similar levels of mortality were achieved by 4–5 days’ post exposure. These results suggest that *M. anisopliae* mycoinsecticide baits may offer an alternative to conventional chemical insecticides for the control of house flies in suitable areas.

## 1. Introduction

House flies, *Musca domestica* L. (Diptera: Muscidae), are medically and agriculturally important pests capable of transmitting a variety of pathogens [1,2,3]. Current control of house flies depends heavily on the use of chemical insecticides [4] though excessive use of these insecticides has led to populations of house flies that are resistant to almost all classes of insecticide used against them [5,6,7]. Concerns regarding the resistance to chemical insecticides and the increasingly negative perception of these chemicals by industry and the general public have led to demand for safer alternatives. Entomopathogenic fungi formulated as mycoinsecticides offer one alternative to conventional chemical insecticides for controlling house flies. Current commercially available mycoinsecticides are based predominately on *Metarhizium anisopliae* (Metschn.) Sorokin (Hypocreales: Clavicipitaceae) or *Beauveria bassiana* (Bals.-Criv) Vuill. (Hypocreales: Cordycipitaceae) conidia as the active ingredient [8]. These fungi are natural insect pathogens and are considered safe to use with minimal environmental or human health risks [9,10]. *Musca domestica* have previously been shown to be highly susceptible to a variety of entomopathogenic fungi and studies have indicated that isolates of *M. anisopliae* can be particularly virulent to these flies [11,12,13,14,15,16].

Current commercially available mycoinsecticides registered for use against house flies are generally formulated as wettable powders or dispersible emulsions for residual or direct application sprays [8] and as such may be unsuitable for areas such as feed stores, animal pens, and residential buildings. Chemical insecticide bait formulations are commonly used for house fly control in animal production facilities [4] with granular “scatter” baits being the most common due to ease of use and target specificity [17]. There are few bait formulations that use entomopathogenic fungi as active ingredients, with only one product, utilising *B. bassiana*, registered for use with house flies in the USA (balEnce™). Currently no *M. anisopliae* based mycoinsecticide is commercially available for the control of house flies. Previous research has shown mycoinsecticides can cause high mortality in adult *M. domestica* when used as bait formulations [18,19,20,21,22,23], with dry baits based on either harvested conidia or conidia production substrates performing better than those first formulated as conidial suspensions and subsequently applied to substrates.

The efficacy of a mycoinsecticide bait formulation for house flies can be highly dependent on the attractive or arrestant properties of the bait. Maximising visitation to and contact time with the bait can aid in the uptake of a lethal dose of the active component [24]. Therefore, many bait formulations contain attractants and phagostimulants. Early research focused on natural products including molasses, milk and yeast [25,26]. The identification and subsequent synthesis of a fly sex pheromone, (Z)-9-tricosene (muscalure) [27,28], as well as the discovery of attractive volatile organic compounds derived from food sources [29], has led to the development of a variety of baits containing proprietary attractants [30]. The current study presents a series of laboratory assays aimed at developing a bait formulation containing a *M. anisopliae* isolate in a dry granule form for use against *M. domestica*.

## 2. Materials and Methods

### 2.1. Insect Cultures

*Musca domestica* used in this study were reared in a controlled environment room at constant conditions of 27 ± 2 °C, 67% ± 5% relative humidity (RH) and a photoperiod of 12:12 (light:dark) hours. Insects were originally obtained from field collections in Southeast Queensland. Larvae were reared on a pollard, alfalfa meal (milled lucerne) and cornmeal medium (2 + 1 + 0.5 by weight and water to moisten). Adult flies were given a protein feed consisting of skim milk powder, pollard and alfalfa meal (2 + 1 + 0.5 by weight, water to moisten) in preparation for egging. Gravid flies were provided with a small dark vial containing approximately 30 g of egg laying medium (1:1 mix of old and new larval medium) for a period of 6 h for oviposition. Approximately 0.7 g of eggs were transferred to a 1 L plastic container containing larval medium to initiate the next generation.

### 2.2. Fungal Isolate and Conidia Production

The *Metarhizium anisopliae* isolates used in this study were taken from the Queensland Department of Agriculture and Fisheries (DAF) entomopathogenic fungal culture collection housed at the Ecosciences Precinct (ESP), Brisbane, Australia. These isolates were originally obtained from soil or insect samples collected in Queensland, maintained on potato dextrose agar (PDA) (Difco™) slopes held at 4 °C and −20 °C. Production of conidia was completed via a biphasic process with a liquid culture grown to inoculate solid media. The liquid culture consisted of sterile yeast peptone broth inoculated with conidia scraped from 14 day-old cultures on Oatmeal agar (Difco™). Liquid cultures were grown for 5 days at 28 °C in an orbital shaker at 120 rpm. Mushroom spawn culture bags containing 1.5 kg sterilised rice were each inoculated with 150 mL of the liquid culture and incubated for seven days at 28 °C on wire racks after which the solid cultures were broken up then incubated for a further 14 days. Bags were then opened and left to air dry for 4–5 days at 19 °C in a dehumidified room.

### 2.3. Isolate Screening

Bioassays were conducted in small containers with gauze lids (70 mm height × 80 mm diameter) inside a controlled environment room (27 °C, 65% RH and 12:12 h light:dark). In each container, flies were provided water in a 25 mL vial with a cotton wick inserted through the lid and 2 g of granulated sucrose in a small plastic lid. In treatment containers, conidial powder was mixed thoroughly with the sucrose at a concentration of 0.0083 g conidia/1 g sucrose (≈3.32 × 10^8^ conidia/gram). Five *M. anisopliae* isolates were tested (Table 1), with three replicates each. Three control containers were prepared similarly with granulated sucrose. Twenty, unsexed, 2–5-day-old flies from the laboratory colony were anaesthetised with CO_2_, placed in each container and held at 27 °C and 65% RH with a 12:12 h light:dark regime. Flies were assessed for mortality every 24 h for 7 days. This assay was repeated for a total of two complete experimental replicates.

### 2.4. Fly Visitation to Baits

*Metarhizium anisopliae* isolate M16 was selected as the best candidate for development of the bait formulation due to both its high virulence to *M. domestica* (see Section 3.1) and the relative ease of mass production of its conidia. The production of *M. anisopliae* M16 was completed using a solid substrate of white rice at the final stage, which enabled granular bait development without the need to harvest conidia. The mycoinsecticide bait formulations used hereafter were formulated using this substrate.

Fly visitation to the bait formulations was assessed in an insect behaviour observation facility (IBOF) (6 × 3.2 m) at ESP (27 °C, 65% RH and 12:12 h light:dark). The formulations consisted of *M. anisopliae* M16 coated rice mixed with different additives thought to have either arrestant or attractive properties. The compositions of the trial bait formulations are outlined in Table 2. For the bait containing (Z)-9-tricosene, 5 g of sterile rice was pretreated with 0.1% (Z)-9-tricosene diluted in hexane. The treated rice was then left to air-dry, allowing the hexane to evaporate. Each of the bait treatments was contained in a 9 cm Petri dish and placed on the floor of the IBOF, spaced 30 cm apart with the location of each bait assigned at random. A high-resolution camera (Canon EOS 700D, 18 MP) fitted with an 18–200 mm lens (SIGMA Corporation) was mounted to a tripod and positioned to photograph all of the baits within a single frame. A timer kit (CAPTUR timer kit, Hähnel Industries Ltd., Melbourne, Australia) was programmed to take 3 photographs at 10 s intervals every 15 min for 3 h once started. Once the baits and camera were placed in position, 300 unsexed, 2–4-day-old adult *M. domestica* were released into the room from a set position 1.5 m away from the baits. The timer protocol for the camera was set to delay for 15 min once activated to allow the flies to recover from the CO_2_ used to anaesthetise them for collection. The number of flies on each bait in each photograph was counted and recorded. This assay was repeated for a total of six complete replicates.

### 2.5. Bait Efficacy

The efficacy of the most promising granule bait from the visitation assay (*M. anisopliae* M16 substrate + skim milk powder) (see Section 3.2) was assessed in a laboratory trial and compared to a commercially available insecticide bait for house flies. Bioassays were conducted in small containers with gauze lids (70 mm height × 80 mm diameter) inside a controlled environment room (27 °C, 65% RH and 12:12 h light:dark). Six replicate bioassay containers were used per bait treatment, with baits contained in small plastic lids. Trial baits included: (1) 1 g sterilised white rice (control), (2) 1 g rice coated in *M. anisopliae*, (3), 1 g rice coated in *M. anisopliae* + 1 g skim milk powder and (4) 1 g Quickbayt^®^ (Bayer CropScience Pty Ltd., Brisbane, Australia) (0.5% imidacloprid + 0.1% (Z)-9-tricosene). Twenty, unsexed, 2–5-day-old flies from the laboratory colony were anaesthetised with CO_2_ and placed in each container and then incubated at 27 °C, 65% RH and 12:12 h light:dark. Flies were assessed for mortality every 24 h for 7 days. This assay was repeated for a total of two complete experimental replicates.

### 2.6. Statistical Analysis

Survival data for the isolate screening assay and the bait efficacy assay were analysed using Kaplan–Meier survival analysis. The overall test of equality of survival distributions and subsequent post hoc pairwise comparisons between groups were made with the log-rank test [31]. Count data for the bait visitation assay were square-root transformed (x + 0.5) to stabilise the variance [32] before analysis by a repeated measures analysis of variance (ANOVA). The Greenhouse–Geisser epsilon correction factor was used to adjust for temporal autocorrelation before the probabilities were calculated. Means presented are back-transformed along with approximate standard errors from the transformed data. Means were compared using Fisher’s least significant differences (LSD). All data analyses were performed using GENSTAT v18 [33].

## 3. Results

### 3.1. Isolate Screening

**Figure 1:**
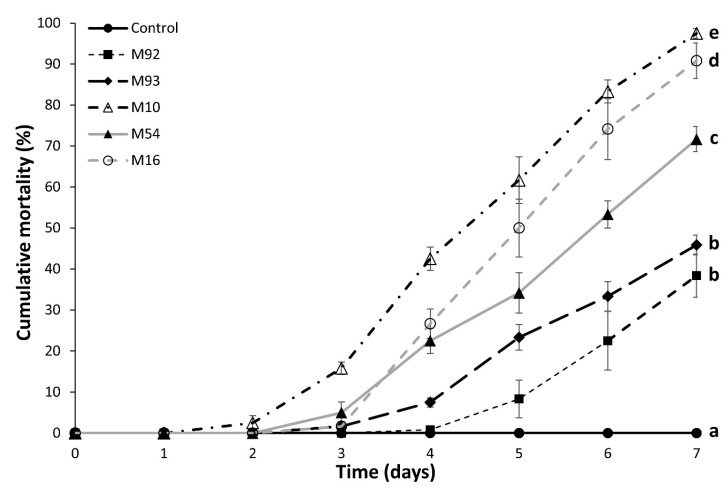
Cumulative mortality (±SE) of *Musca domestica* following exposure to different isolates of *Metarhizium anisopliae* at a dose of approximately 6.64 × 10^8^ conidia. Survival curves of isolates labelled with the same letter are not significantly different.

Survival distributions of *M. domestica* exposed to the selected *M. anisopliae* isolates were significantly different to the control (χ^2^ = 377.46, d.f. = 5, *p* < 0.001), with all isolates causing over 35% mortality by day 7 (Figure 1). The virulence of the selected isolates varied substantially, with all isolates except M92 and M93 causing significantly different levels of mortality. M10 and M16 demonstrated the highest virulence to *M. domestica* with both causing over 90% mortality by day 7. Notably, the *M. anisopliae* isolates originally sourced from *M. domestica* field collections (M54, M92 and M93) had significantly lower virulence than the others.

### 3.2. Fly Visitation to Baits

**Figure 2:**
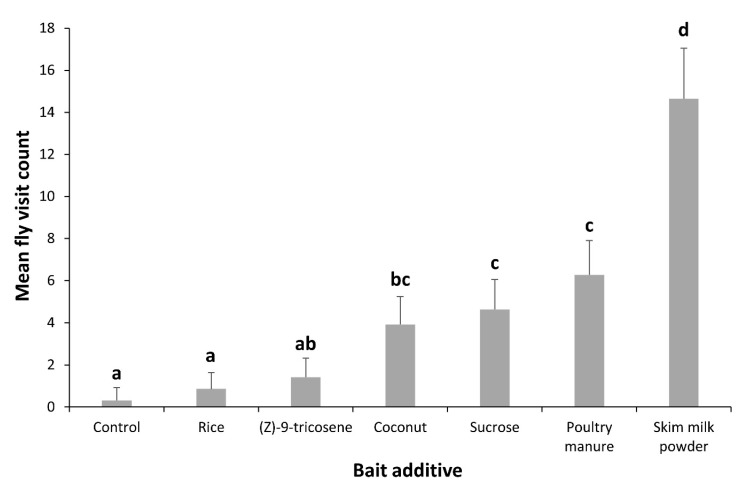
Mean fly visitation (+SE) to trial bait formulations containing *Metarhizium anisopliae* M16 conidia mixed with various additives. Fly visit counts were made at 15 min intervals over a 3 h period. Means labelled with the same letter are not significantly different.

The effect of bait formulation on fly visitation was significant (F_6, 35_ = 11.9, *p* < 0.001) (Figure 2). Fly visitation to bait formulations was found to differ significantly with time, both overall (F_11, 385_ = 3.33, *p* < 0.05) and between baits (F_66, 385_ = 2.32, *p* < 0.01). The bait containing skim milk powder had significantly higher visitation than any other bait with an average visit count of 14.65 ± 2.40, per sample time. The baits containing coconut, sucrose or poultry manure also had significantly higher visitation than the control with average counts of 3.91 ± 1.34, 4.63 ± 1.43 and 6.27 ± 1.63, per sample time, respectively. The bait containing the conventional fly attractant, (Z)-9-tricosene, demonstrated no significant level of attraction when compared to the control. Fly visits to the bait containing skim milk powder peaked at 15 min into the test period, and tended to decrease thereafter. Baits containing coconut, sucrose or poultry manure showed a general increase in fly visitation over time.

### 3.3. Bait Efficacy

**Figure 3:**
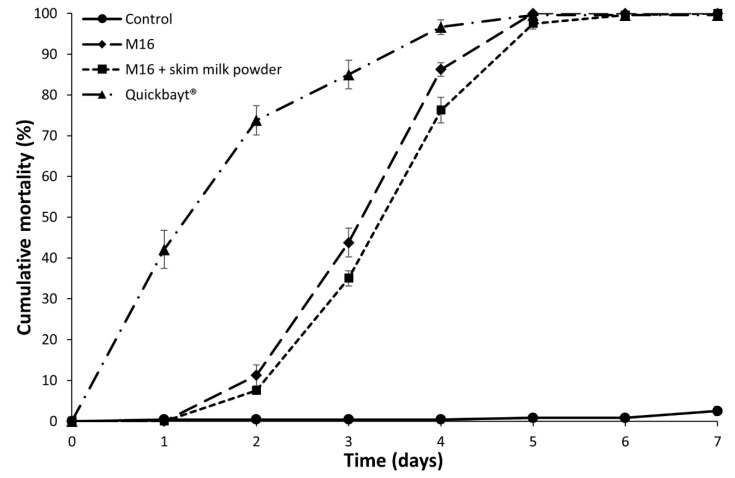
Cumulative mortality (±SE) of *Musca domestica* following exposure to a commercial imidacloprid fly bait (Quickbayt^®^) and mycoinsecticide baits containing *Metarhizium anisopliae*. All survival curves are significantly different.

Survival distributions of *M. domestica* exposed to the granule baits were significantly different (χ^2^ = 971.93, d.f. = 3, *p* < 0.001) (Figure 3). All bait formulations caused significantly higher mortality than the control and mortality differed significantly between all bait formulations. The commercial granule bait containing 0.5% imidacloprid (Quickbayt^®^) demonstrated the highest efficacy, reaching 50% mortality after two days’ exposure. The baits containing *M. anisopliae* both achieved 50% mortality between 3–4 days’ exposure. Exposure to all treatment baits caused over 99% mortality of *M. domestica* by the conclusion of the assay at day 7.

## 4. Discussion

Mycoinsecticides in bait formulation offer an alternative to conventional chemical insecticides for the control of house flies in areas where the spraying of insecticides is undesirable. The current study presented a series of laboratory assays aiming to develop a bait formulation utilising a virulent isolate of *M. anisopliae*. Five isolates of *M. anisopliae* obtained from varying sources were selected for screening against adult *M. domestica* based on origin and previous conidia production data. Isolates M10 and M16 were the most virulent causing 97.5% and 90.8% mortality by seven days’ post exposure (Figure 1). Although M10 was more virulent than M16, M10 conidia proved difficult to mass produce, and subsequently, M16 was selected for further development due to both its virulence and ease of mass production of conidia. Notably, the isolates originally sourced from field-sampled house flies M54, M92 and M93, were significantly less effective at killing flies at the dose tested than the two strains isolated from soil. This suggests a level of attenuation by the *M. domestica* host-isolated strains, though previous studies have demonstrated no relationship between the virulence potential of an isolate and its source [34,35]. This does, however, highlight the need to screen a variety of fungal isolates against target pests when considering the development of a mycoinsecticide.

The *M. anisopliae* isolates used in this study were grown on a solid rice substrate as the final stage of mass production. The granular nature of this substrate lends itself to the development of a bait formulation without the need for the harvesting of conidia. White rice substrate coated in *M. anisopliae* M16 conidia was mixed with a variety of additives thought to be attractive or act as feeding stimulants to adult house flies. Fly visitation to the trial bait formulations indicated that skim milk powder was the most effective additive for increasing fly contact with the bait (Figure 2). Powdered milk is a high-quality protein source which has been extensively used in laboratory rearing methods for house flies and subsequently incorporated into a bait in other studies [16,18]. Powdered milk has also been shown to act as a UV protectant when mixed with conidia [24], increasing its usefulness as an additive. Baits containing air-dried coconut meat, granulated sucrose, and composted poultry manure also had significantly higher fly visitation than the control and visitation to these baits tended to increase over time. The general increase in visitation with older baits may be attributed to the “fly factor” whereby substances previously fed on by conspecifics are visited more [36,37]. Sugar is a commonly used additive for both laboratory assays and commercial baits so the results seen here were as expected [18,20,23]. Fly attraction to the bait containing the pelletised poultry manure is likely due to an attraction to ammonia [29,38], however, future work analysing the volatile profile of this product may yield new insights. (Z)-9-tricosene, the fly attractant predominant in commercially available baits, produced no significant response as an additive. Previous studies have concluded that the addition of (Z)-9-tricosene as an attractant in bait formulations may not necessarily increase house fly activity and that other components may be more important for attracting flies [39]. It was noted that although the hexane used to dilute the (Z)-9-tricosene was allowed to evaporate from the rice, conidia from the sporulated substrate clearly adhered to the treated rice after they were combined. This may have inhibited the attractive properties of the (Z)-9-tricosene. Fly visitation to the bait formulations was assessed by making instantaneous counts of the number of flies on a bait over successive intervals. Therefore, distinguishing between additives that were functioning as attractants or arrestants based on these data was not possible. This method of measuring fly visitation is common in the literature [40,41,42] and is sufficient for determining whether or not an additive could increase the effectiveness of the bait, however, the nature of the behavioural response caused by the additive can have serious implications for field application and should be investigated.

The efficacy of the bait formulation of *M. anisopliae* M16 with the addition of skim milk powder was compared to a commercially available insecticide bait and a positive control. Quickbayt^®^ is a granular bait containing 0.5% imidacloprid and a proprietary attractant blend that includes 0.1% (Z)-9-tricosene. All three treatment baits caused over 99% mortality of adult house flies after seven days of exposure, however, Quickbayt^®^ demonstrated the fastest kill time at 50% mortality by day 2 (Figure 3). The level of mortality achieved by the mycoinsecticide baits reached comparable levels to the Quickbayt^®^ between 4–5 days. The long incubation period required to achieve mortality with entomopathogenic fungi potentially allows infected flies to continue to reproduce before death. Previous studies have demonstrated, however, that *M. anisopliae* infection in house flies can significantly reduce female fecundity and that *M. anisopliae* is readily transmissible between conspecifics [43,44,45]. Thus, the use of mycoinsecticides can have additional population reducing effects aside from direct mortality. The level of mortality achieved by the mycoinsecticide bait is consistent with other studies. Geden, Rutz and Steinkraus [18] mixed sugar and powdered milk with *B. bassiana* conidia and demonstrated 90% mortality in adult house flies by day 7. Renn, Bywater and Barson [19] achieved 95–100% mortality with an *M. anisopliae* sugar bait by day 10. However, direct comparisons between studies testing entomopathogenic fungi are difficult due to differences in dose, exposure time, conidial quality, isolate and fly susceptibility.

## 5. Conclusions

This study developed and evaluated mycoinsecticide bait formulations for the control of house flies. The results demonstrated that similar levels of mortality can be achieved by mycoinsecticide baits when compared to a chemical insecticide product and that visitation to the mycoinsecticide bait formulations by flies can be increased with the addition of components demonstrating attractive or arrestant properties. The final bait formulation consisting of *Metarhizium anisopliae* conidia-coated rice mixed with skim milk powder achieved 50% mortality in house flies by 3–4 days after exposure. This result suggests that it may be possible to achieve effective control of house fly populations in areas suitable for bait deployment. Future development of this bait formulation should include the optimisation of the amount of milk powder additive and further testing of other known attractants such as the components of the proprietary blends used in available products.

## Figures and Tables

**Table 1:** Original source and geographic origin of *Metarhizium anisopliae* isolates collected in Queensland, Australia, and used in this study.

Isolate No.	Queensland Plant Pathology Herbarium (BRIP) Collection No.	Original Host/Source	Geographic Origin and Year of Isolation
**M10**	42,411	Soil	South Johnstone, 1999
**M16**	42,412	Soil	Aratula, 1999
**M54**	61,277	*Musca domestica*	Dalby, 2002
**M92**	not submitted	*Musca domestica*	Irvingdale, 2013
**M93**	not submitted	*Musca domestica*	Irvingdale, 2013

**Table 2:** Composition of trial bait formulations containing *Metarhizium anisopliae* M16 and various additives.

Bait	Rice Substrate	Additive
**Control**	10 g sterile white rice	none
**1**	5 g M16 coated white rice	5 g sterile white rice
**2**	5 g M16 coated white rice	5 g sterile white rice containing 0.1% (Z)-9-tricosene
**3**	5 g M16 coated white rice	5 g air dried coconut meat
**4**	5 g M16 coated white rice	5 g granulated sucrose
**5**	5 g M16 coated white rice	5 g composted poultry manure pellets
**6**	5 g M16 coated white rice	5 g skim milk powder
